# First Detection of Boosepivirus B1 in a Sick Yearling's Nasal Swab, Mexico

**DOI:** 10.1111/irv.70165

**Published:** 2025-09-28

**Authors:** Judith U. Oguzie, Gustavo Hernandez‐Vidal, Gustavo Moreno‐Degollado, Gregory C. Gray

**Affiliations:** ^1^ Division of Infectious Diseases, Department of Internal Medicine University of Texas Medical Branch Galveston Texas USA; ^2^ Faculty of Veterinary Medicine Universidad Autónoma de Nuevo León Escobedo Nuevo León Mexico; ^3^ Department of Microbiology and Immunology University of Texas Medical Branch Galveston Texas USA; ^4^ Institute for Human Infections and Immunity University of Texas Medical Branch Galveston Texas USA

**Keywords:** boosepivirus, Mexico, nasal swab, sick yearling

To the Editor,

1

Boosepivirus (BooV), a recently proposed genus within the family *Picornaviridae*, includes species A, B, and C, each with distinct genotypic subdivisions (e.g., B1 and B2) [1]. To date, BooV has been detected exclusively in fecal samples from cattle in several geographic locations with no prior reports of its presence in respiratory specimens [2, 3, 4, 5]. Here, we describe the first detection of the BooV B1 genotype in a nasal swab collected from a beef yearling exhibiting respiratory symptoms in Mexico.

As part of a One Health surveillance for novel respiratory viruses on US and Mexican beef cattle farms in 2024, farm workers collected deep nasal swabs from 40 sick beef cattle and 12 bioaerosol samples from a farm in Nuevo León, Mexico. RNA was extracted from nasal swab specimens using the QIAamp Viral RNA Mini Kit on the QIAcube Connect automated system (QIAGEN Inc., Valencia, CA). Sequencing libraries were prepared with the Illumina Nextera XT Library Prep Kit (San Diego), following established protocols [6]. Libraries were sequenced on the Illumina NovaSeq X platform, generating 75‐bp paired‐end reads.

Raw metagenomic sequencing (mNGS) reads were processed using the Chan Zuckerberg ID (CZ ID) platform (https://czid.org). Subsequently, BooV genomes were aligned with published BooV sequences from the NCBI database using MAFFT [7], and a maximum likelihood phylogenetic tree was generated with IQ‐TREE [8]. The resulting tree was visualized and annotated in FigTree v1.4.4 (http://tree.bio.ed.ac.uk/software/figtree/).

One of the 40 sick cow samples from the farm was chosen for mNGS based on pan‐coronavirus assay positivity, which yielded evidence for a rodent coronavirus [9]. The specimen was collected from a 17‐month‐old beef yearling with fever and nasal discharge. The specimen had no molecular evidence of influenza A or D.

We assembled a contig of 7512 nt in length. BLASTn analysis demonstrated 87.63% nucleotide identity with strain 21‐0305 (GenBank accession no. MZ052226.1). At the protein level, BLASTp analysis of the amino acid sequence revealed 98.33% identity with strain Bo‐12‐11/2009/JPN (GenBank accession no. LC036581.1) and 98.29% identity with strain 21‐0305 (GenBank accession no. MZ052226.1). Phylogenetic analysis further showed that our sequence formed a monophyletic cluster with MZ052226.1, which was previously isolated from a diarrheic calf in the United States (Figure 1).

**FIGURE 1:**
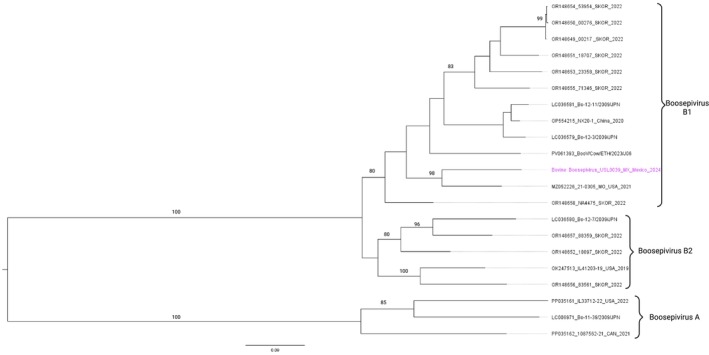
Maximum‐likelihood phylogenetic tree of boosepivirus sequences. The tree was constructed using complete or near‐complete polyprotein gene sequences available in GenBank and the sequence generated in this study (highlighted in purple). The analysis was performed using the IQ‐TREE software under the best‐fit substitution model, and branch support values were calculated with 1000 bootstrap replicates. The scale bar indicates nucleotide substitutions per site. Strain names, accession numbers, host species, and countries of origin are shown where available. Our sequence clustered within the boosepivirus B1 lineage, closely related to strains previously detected in the United States.

Notably, this is the first report of BooV detection in a nasal specimen. The BooV sequence has been submitted to the GenBank database under accession number PX262393. Although we cannot rule out fecal contamination of the yearling's airway, as the yearling had marked respiratory signs, our finding suggests the possibility of BooV's association with respiratory illness in cattle. This detection highlights the value of employing broad‐range diagnostics when evaluating respiratory specimens from symptomatic cattle.

## Author Contributions

**Judith U. Oguzie:** conceptualization, investigation, methodology, writing – original draft, writing – review and editing, visualization, validation, formal analysis. **Gustavo Hernandez‐Vidal:** investigation, methodology, writing – review and editing. **Gustavo Moreno‐Degollado:** investigation, methodology, writing – review and editing. **Gregory C. Gray:** conceptualization, methodology, investigation, formal analysis, supervision, funding acquisition, project administration, resources, writing – review and editing.

## Conflicts of Interest

The authors declare no conflicts of interest.

## Data Availability

The data that support the findings of this study are available from the corresponding author upon request.
